# External Urethral Orifice Metastasis of Cervical Cancer Treated With Intraluminal Urethral Brachytherapy Using a Lumencath Applicator: The First Case Report

**DOI:** 10.1016/j.adro.2021.100828

**Published:** 2021-10-06

**Authors:** Yoshiaki Takagawa, Sachiko Izumi, Tomoyuki Okano, Eiichi Takahashi, Yuki Wakamatsu, Megumi Takahara, Haruka Okada, Midori Kita

**Affiliations:** a Department of Radiation Oncology, Southern Tohoku Proton Therapy Center, Fukushima, Japan; b Departments of Radiology, Tokyo Metropolitan Tama Medical Center, Tokyo, Japan; c Departments of Obstetrics and Gynecology, Tokyo Metropolitan Tama Medical Center, Tokyo, Japan; d Departments of Pathology, Tokyo Metropolitan Tama Medical Center, Tokyo, Japan

## Introduction

Cervical cancer is the fourth most common malignancy in women worldwide, with more than 300,000 deaths reported in 2018.^1^ The staging of cervical cancer is obtained by combining the findings of physical examination, endoscopic procedures (hysteroscopy, cystoscopy, and proctoscopy), and imaging modalities according to the International Federation of Gynecology and Obstetrics (FIGO) guidelines.^2^ These guidelines also indicate that suspicious lesions should be confirmed by biopsy. Recently, definitive chemoradiotherapy has played a more important role in the treatment of patients with locally advanced cervical cancer. Physical examination is the most important part of cervical cancer staging for both gynecologists and radiation oncologists. We encountered a rare case of isolated external urethral orifice metastasis of primary cervical cancer, where we achieved excellent local control for both primary tumor and external urethral orifice metastasis when performing high dose-rate brachytherapy (HDR-BT) using a Lumencath applicator. Therefore, we report herein the first case of external urethral orifice metastasis of primary cervical cancer with a literature review.

## Case Presentation

Written informed consent was obtained from the patient for publication of this case report and accompanying images.

**Figure 1:**
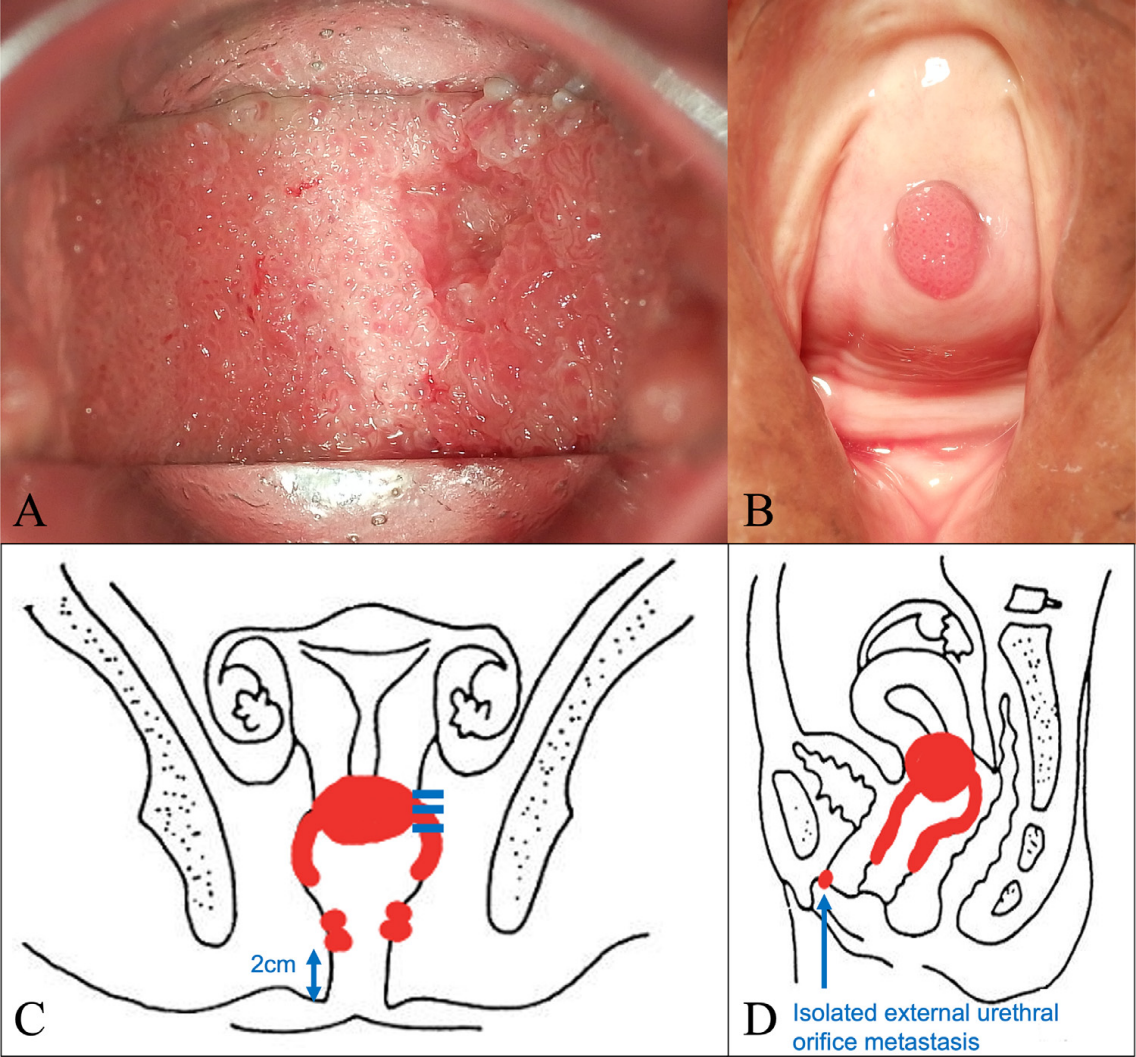
Pretreatment gross findings of the cervix (A) and the external urethral orifice (B). There were similar gross tumors in the cervix and the external urethral orifice. Coronal view (C) and sagittal view (D) of schema of the primary tumor. It involved the lower third of the vagina.

**Figure 2:**
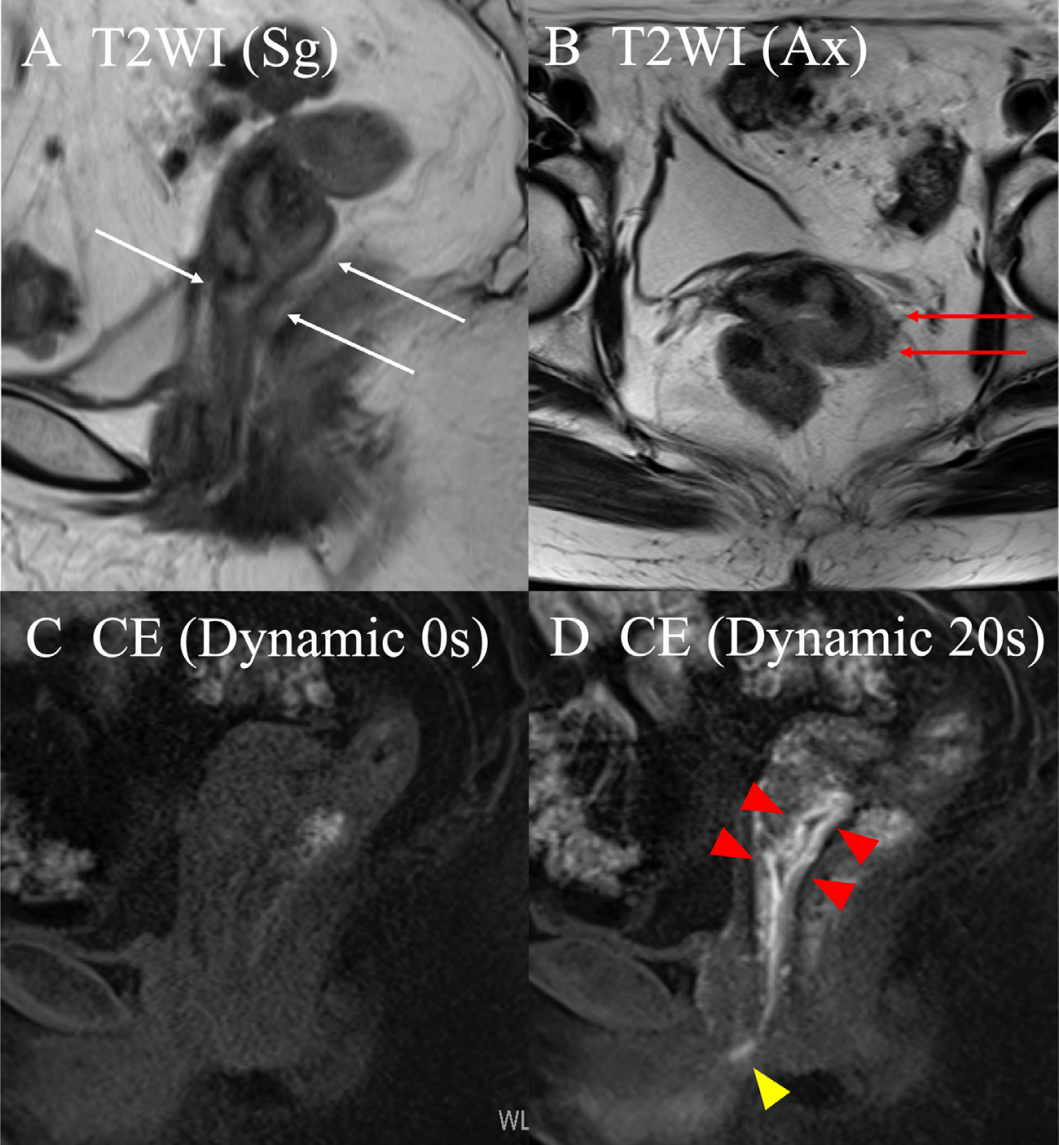
Pretreatment magnetic resonance imaging (MRI) findings of the cervix, vagina, and external urethral orifice. Sagittal (A) and axial (B) T2-weighted image of the cervix show tumor- extended vagina (white arrow) and small left parametrium invasion (red arrow). Predynamic enhanced sagittal T1-weighted image (C) and 20-seconds dynamic image (D) show early contrast effect in the cervix and vagina (red arrowhead) and external urethral orifice (yellow arrowhead).

**Figure 3:**
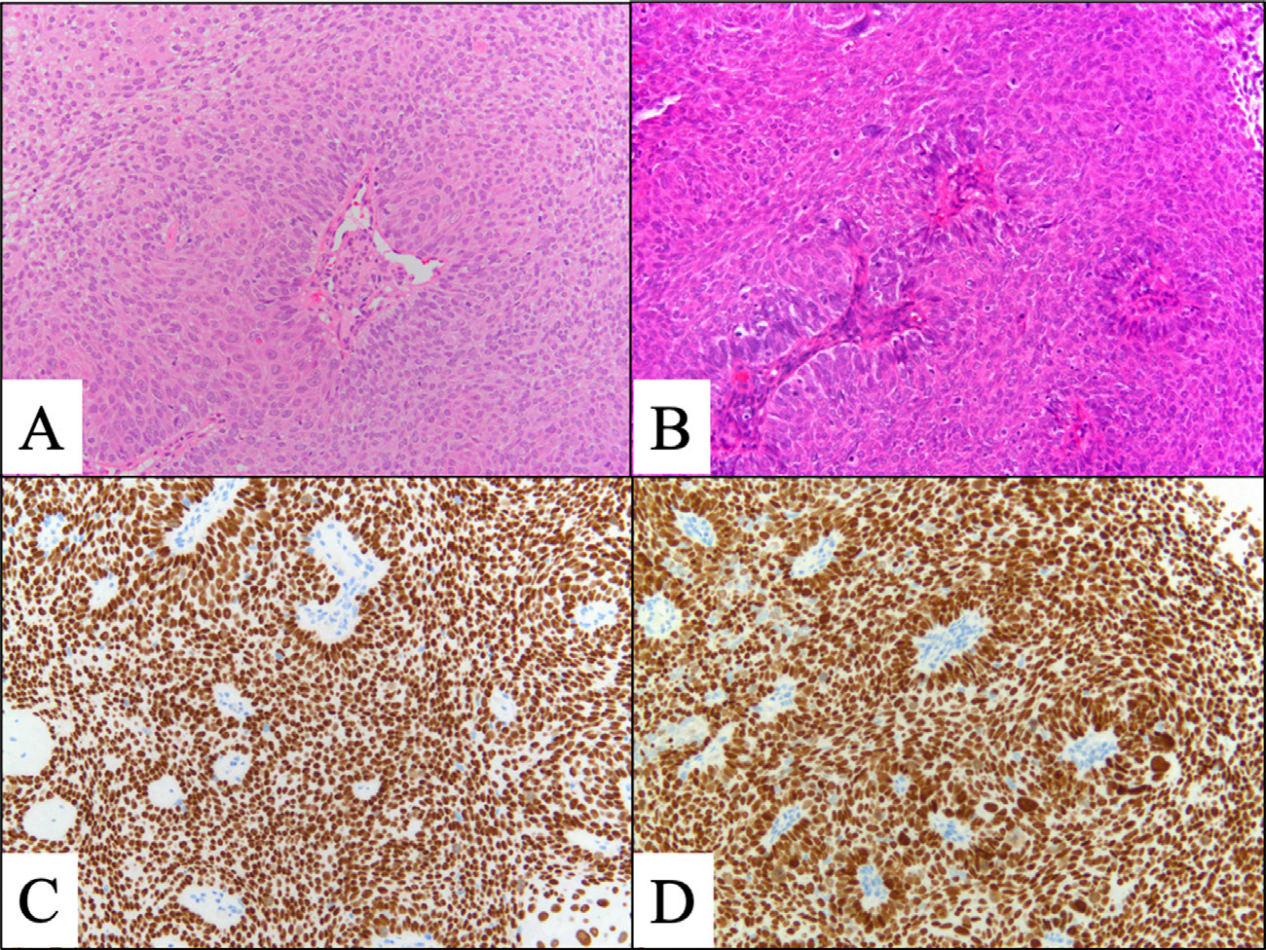
Hematoxylin and eosin-stained sections at 20 × magnification of the cervical tumor (A) and the external urethral orifice tumor (B) showing atypical cells with rounded swollen nuclei proliferating in alveolar habits. Cervical tumor indicating squamous cell carcinoma and external urethral orifice tumor suggesting metastatic squamous cell carcinoma. P40 immunostaining of specimens at 20 × magnification of the cervix (C) and the external urethral orifice (D) is positive, and they have a similar staining pattern.

**Figure 4:**
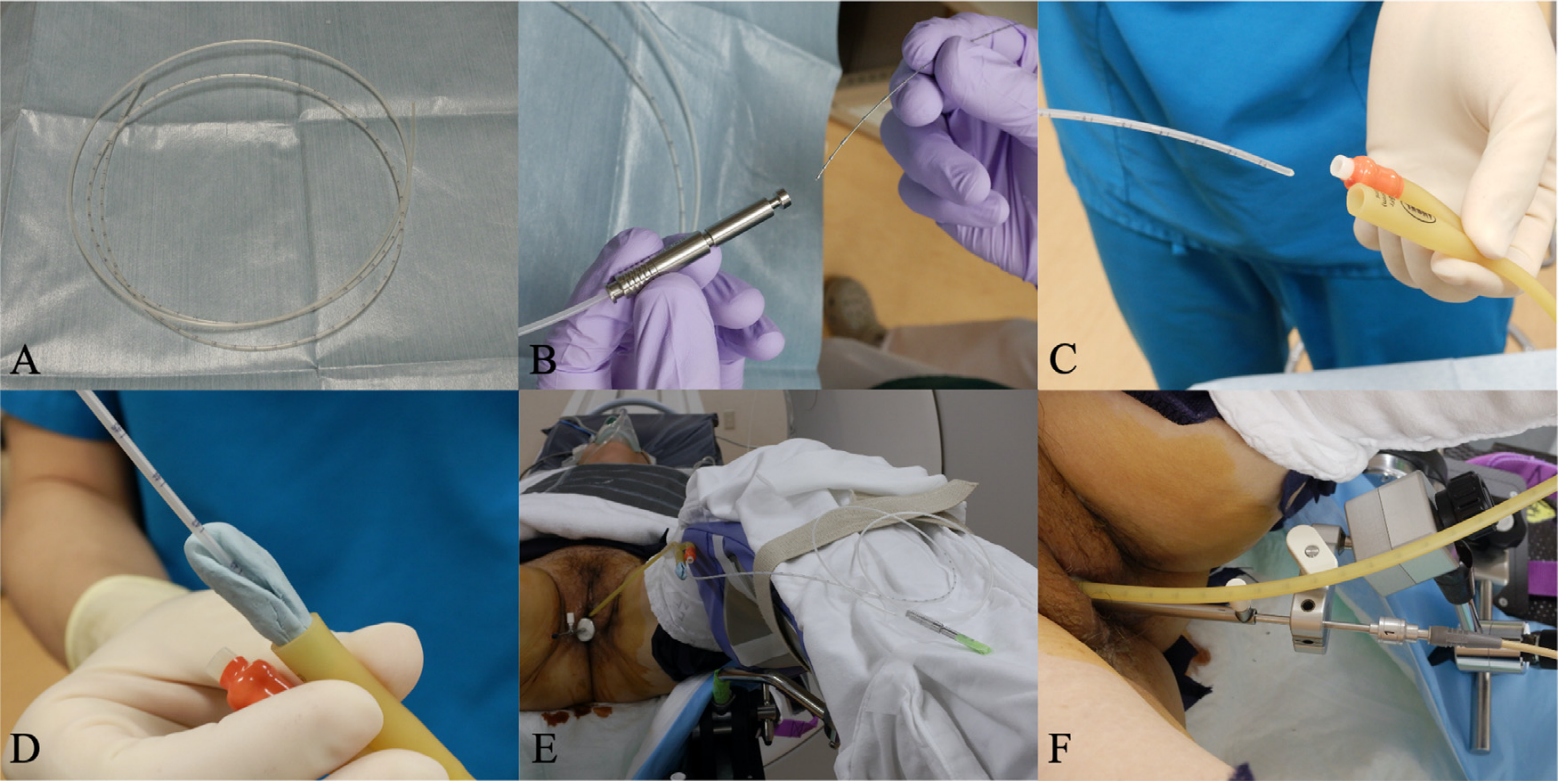
Photographs of the procedure of intraluminal urethral brachytherapy (BT). (A) Overall image of the Lumencath applicator. (B) Dummy source inserted into the Lumencath applicator. (C) The Lumencath applicator with dummy source inserted into the 16-French Foley catheter. (D) Image of the hollow cap made of the dental vinyl silicone impression material. (E, F) Photographs of the applicator insertion and immobilization during BT.

**Figure 5:**
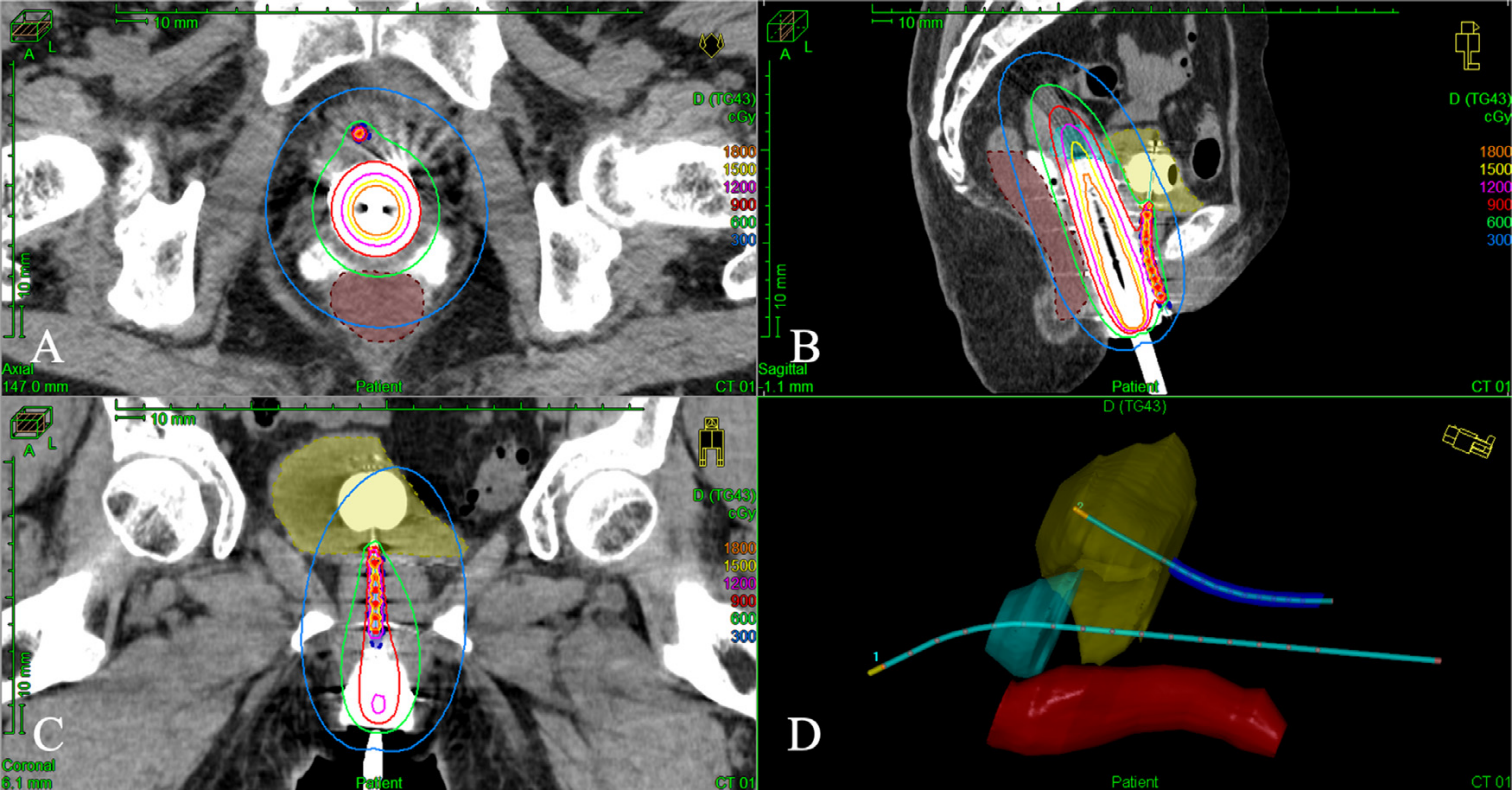
Dose distribution and applicator reconstruction of computed tomography (CT)-based image guided brachytherapy. (A) Axial view, (B) sagittal view, and (C) coronal view of treatment planning. (D) Three-dimensional image of the applicator reconstruction. Light blue shade, clinical target volume of cervix; yellow shade, bladder; brown shade, rectum; blue shade, urethra. Green line represents 100% (6 Gy) isodose line. The red line represents the 150% (9 Gy) isodose line.

**Figure 6:**
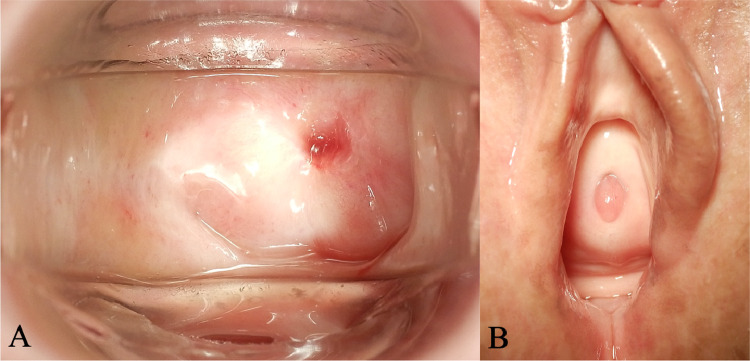
Posttreatment gross findings of the cervix (A) and the external urethral orifice (B) 9 months after chemoradiotherapy. Tumors in the cervix, vagina, and external urethral orifice have completely disappeared.

A 78-year-old woman presented with a small amount of genital bleeding. Cervical squamous cell carcinoma (SCC) was diagnosed. The primary tumor was observed in the cervix (Fig 1A), which extended the vagina circumferentially and involved the lower third of the vagina (Fig 1C,D). The length between the caudal edge of the vaginal lesion and entrance of the vagina was 2 cm. Small left parametrial involvement, which did not extend to the pelvic wall, was revealed by gynecologic examination. Computed tomography (CT), magnetic resonance imaging (MRI), and positron emission tomography/CT were performed. Pretreatment MRI images are shown in Figure 2. Cervical SCC stage IIIA was diagnosed based on the FIGO 2018 staging, and definitive chemoradiotherapy was planned. At the time of consultation with the radiation oncology department, we noticed swelling of the external urethral orifice, which showed gross findings similar to those of the cervix and vagina (Fig. 1B). No abnormal findings were observed in the vulva or clitoris. In addition, no urologic symptoms (urination pain, hematuria, dysuria) were observed. We performed a biopsy of the external urethral orifice, and metastatic SCC was histologically diagnosed (Fig 3). P40 immunostaining of the specimen of the primary tumor and external urethral orifice was performed. Both specimens were positive for p40 immunostaining and the same staining pattern (Fig 3). As a result, external urethral orifice metastasis of the cervical cancer was diagnosed. Based on the clinical findings, we reviewed MRI findings again, and found an early enhanced effect on the external urethral orifice similar to the cervix and vagina (Fig 2C,D). Therefore, we considered that it may express a metastatic lesion. The patient's clinical stage was T3aN0M1, IVB (TNM classification, 8th edition). However, we did not perform cystoscopy or urinary cytology. The pretreatment SCC antigen level was 6.5 ng/mL. We planned definitive chemoradiotherapy with weekly cisplatin (40 mg/m^2^) and external beam radiation therapy (EBRT) followed by HDR-BT. The EBRT dose was 50.4 Gy delivered in 28 fractions for the whole pelvis and vulva. We used the conventional 4 field box irradiation up to 30.6 Gy. A midline block (3 cm width at the isocenter) was inserted into the treatment field (anterior-posterior/posterior-anterior field) after delivering 30.6 Gy/17 fractions to the whole pelvis and vulva. After 30.6-Gy irradiation, we added weekly definitive HDR-BT. We performed HDR-BT immediately after an interim pelvic MRI and gynecologic examination. As a result, the primary tumor, including left parametrium involvement and external urethral orifice metastasis, were shrunken. We considered that there was no need for an interstitial needle because of the tumor involvement. Therefore, we used the Tandem and Cylinder (Elekta, Sweden) and Lumencath applicators (Nucletron Operations BV, Veenendaal, the Netherlands) to treat not only the cervix and vagina but also the entire urethra in the HDR-BT sessions. The diameter of the Lumencath applicator was 6 French with a length of 150 cm (Fig 4A). For remote after-loading, we used microSelectron HDR-V3 with Oncentra Brachy (Elekta) with Ir-192. We performed CT-based image guided brachytherapy (BT) in every HDR-BT session. The procedure for intraluminal urethral HDR-BT is shown in Figure 4. First, we inserted a dummy source into the Lumencath applicator. Second, a Lumencath applicator with a dummy source was inserted into a 16-French, 2-way Foley catheter. The Lumencath applicator was inserted into the end of the Foley catheter. We created a hollow cap of the Foley catheter using a putty-type dental vinyl silicone impression material (GC, Japan) to prevent urinary backflow and fix the Lumencath applicator with the Foley catheter. Third, we inserted the 16-French Foley catheter with the Lumencath applicator into the urethra. After dilation of a fixation balloon in the bladder to immobilize the Foley catheter's depth, we pulled the fixation balloon to the bladder neck and fixed it. Finally, we inserted the Tandem and Cylinder applicators into the cervix and vagina. Pulling and fixing the Foley catheter on the patient's leg, there was no misalignment of the Foley catheter's depth during planning CT and HDR-BT treatment. We delineated the clinical target volume (CTV) for the cervix (CTV_cervix_), vagina (CTV_vagina_), and urethra (CTV_urethra_). The prescribed HDR-BT dose was 24 Gy in 4 fractions. The CTV_cervix_ was contoured based on the The Groupe Européen de Curiethérapie and the European SocieTy for Radiotherapy & Oncology (GEC-ESTRO) recommendation.^3^^,^^4^ Considering tumor involvement, the CTV_vagina_ was contoured by the entire vagina with a 5-mm margin from the surface of the Cylinder applicator, and the CTV_urethra_ was contoured by the entire urethra with a 2-mm margin from the surface of the Foley catheter from the external urethral orifice to the bladder neck. Urethral toxicity was also assessed by using the same definition of the CTV_urethra_. The dose distribution of HDR-BT is shown in Figure 5. The average CTV D90 of the cervix, vagina, and urethra in HDR-BT sessions was 7.3, 6.0, and 5.6 Gy, respectively. The average of the urethra in D0.1 cm^3^ and D1 cm^3^ were 18.2 and 10.3 Gy, respectively. The total biologically equivalent doses in 2 Gy fractions (EQD2) of EBRT (30.6 Gy/17 fractions) plus HDR-BT (24 Gy/4 fractions) based on the linear-quadratic model for CTV D90 of the cervix, vagina, and urethra were 72.5, 62.5, and 59.8 Gy, respectively, assuming an α/β ratio of 10. Total EQD2 for urethra D0.1 cc and D1 cc were 340 and 141 Gy, respectively, assuming an α/β ratio of 3. Weekly cisplatin was administered for 5 courses. As a result, we achieved excellent local control for both primary tumor and external urethral orifice metastasis of cervical cancer (Fig 6). Posttreatment SCC antigen level decreased to within the normal range. Acute toxicities included grade 1 cystitis, urethritis, and grade 2 dermatitis of the vulva and diarrhea. No grade ≥3 acute toxicities were observed. Late toxicity was only grade 1 frequent urination (which existed before therapy and did not worsen after treatment). No tumor recurrence or grade ≥2 late toxicities were observed during the 15-month follow-up.

## Discussion

Primary urethral tumors are rare. The Surveillance, Epidemiology, and End Results study has reported that the annual age-adjusted incidence rates of primary urethral tumors are 4.3/million in men and 1.5/million in women in the United States.^5^ Histologically, transitional cell carcinoma is the most common type (55%), followed by SCC (21.5%) and adenocarcinoma (16.4%). In addition, urethral metastatic tumors are rare.^6^ There are only a few reports of urethral metastatic tumors of urologic and colorectal carcinomas.

On the contrary, locally advanced cervical cancer often involves the vagina. However, cutaneous metastasis, including the vulva arising from cervical cancer, is also rare.^7^^,^^8^ Only a few cases of vulvar skin metastasis of cervical cancer have been reported in the Korean literature.^9^, ^10^, ^11^, ^12^ In these cases, vulvar skin metastasis is observed as a recurrence after the initial treatment. The interval between the diagnosis of cervical cancer and that of cutaneous metastasis was 8 to 131 months. The main clinical manifestations were erythematous papules, nodules, and vesicles in the vulva.

In addition, we found only 6 case reports of clitoral metastasis of cervical cancer.^13^, ^14^, ^15^, ^16^, ^17^, ^18^ The main manifestations of clitoral metastasis are clitoral pain and enlargement. However, in our case, no metastatic lesions were observed in the vulva or clitoris. To the best of our knowledge, the present case is the first report of external urethral orifice metastasis in primary cervical cancer. The external urethral orifice can easily be overlooked on physical examination of patients with cervical cancer, not only by gynecologists, but also by radiation oncologists. Moreover, in the present case, there were no urologic symptoms (urination pain, hematuria, dysuria). Therefore, this rare case report suggested that gynecologists and radiation oncologists should keep in mind to examine not only the cervix and vagina but also the external urethral orifice for patients with primary cervical cancer.

Considering tumor involvement, we performed EBRT followed by intraluminal urethral HDR-BT using the Lumencath applicator to treat the entire urethra. Recently, guidelines on primary urethral carcinoma published by the European Association of Urology have described the role of radiation therapy.^19^ Milosevic et al^20^ reported 34 women with primary urethral carcinoma treated with radiation therapy. Twenty patients (59%) received BT with or without EBRT. The 7-year actuarial overall and cause-specific survival rates were 41% and 45%, respectively. Large primary tumor bulk and treatment with EBRT alone (no BT) were independent poor prognostic factors for local tumor recurrence. In their study, BT reduced the risk of local recurrence by a factor of 4.2.

The largest retrospective study of treating primary carcinoma of the female urethra with radiation therapy was published by the University of Texas M.D. Anderson Cancer Center.^21^ Eighty-six patients received radiation therapy alone: 35 were treated with a combination of EBRT and interstitial BT, 21 received EBRT only, and 30 received interstitial BT only. The cumulative doses ranged from 40 to 106 Gy (median, 65 Gy). The 1-, 2-, and 5-year local control rates in 84 evaluable patients were 72%, 65%, and 64%, respectively. Of note, pelvic toxicity in patients achieving local control was considerable (49%), including urethral stenosis (n = 11), fistula or necrosis (n = 10), and cystitis and/or hemorrhage (n = 6), with 30% of the reported complications graded as severe. Higher doses correlated with a greater incidence of complications, but not with improved local control.

There are few studies of female urethral dose constraints with HDR-BT in the literature. Rajagopalan et al^22^ reported 16 patients with vaginal cancer near/involving the urethra who were treated with HDR interstitial BT. They concluded that patients who receive 5 fractions higher than the 5 Gy/fraction to 0.1 cc of urethra (estimated EQD2 of 85 Gy) are at increased risk of sever urethral toxicity. More recently, Cozma et al^23^ published information about the relationship between urethral dose and genitourinary toxicity among patients receiving HDR interstitial BT. They described that receiver operator curves of urethra D0.1, D0.2, and D0.5 cc were associated with the development of toxicity at total EQD2 >78, >71, and >62 Gy, respectively. In comparison to our case, EQD2 of organ at risk (OAR)_urethra_ D0.1 and D1 cm^3^ were 340 and 116 Gy, respectively. However, these urethral dose constraints were based on the technique of percutaneous interstitial BT approach for vaginal tumor. Because we used intraluminal urethral HDR-BT, these dose constraints were not appropriate for our case.

There are only 2 case reports on the use of a Lumencath applicator in intraluminal BT for treating the male urethra. Chakrabarti et al^24^ reported complete tumor remission and no severe late toxicity using a fraction dose of 7 Gy in 7 weekly fractions prescribed at 5 mm from the Lumencath applicator. Lewis et al^25^ demonstrated intraluminal HDR-BT prescribed with 36 Gy in 9 fractions followed by a boost of 24 Gy in 6 fractions. They also showed good local control and no posttreatment toxicity. However, to the best of our knowledge, this is the first reported case using the Lumencath applicator in intraluminal HDR-BT for treating a female urethra. During the HDR-BT planning, we did not prescribe a 150% dose (9 Gy per fraction) to the CTV_urethra_, 2-mm margin from the surface of the Foley catheter, which is nearly equal to the urethral mucosa, to minimize urethral toxicity. However, there were no grade ≥3 acute or late toxicities in the short-term follow-up, and we achieved excellent local control for both primary tumor and external urethral orifice metastasis of cervical cancer. Intraluminal urethral HDR-BT using the Lumencath applicator is a good treatment option for cervical cancer with urethral involvement. Further follow-up is needed to determine the late toxicities of this treatment.

## Conclusions

This is the first case report of external urethral orifice metastasis of primary cervical cancer treated with intraluminal urethral HDR-BT using the Lumencath applicator. In short-term follow-up, intraluminal urethral HDR-BT using the Lumencath applicator was found to be a good treatment option for cervical cancer with urethral involvement.
